# Effects of sepiolite addition to acrylic-latex paint on pull-off adhesion strength in nanosilver-impregnated and thermally-modified beech (*Fagus orientalis* L.) wood

**DOI:** 10.1038/s41598-024-54451-9

**Published:** 2024-02-20

**Authors:** Hamid R. Taghiyari, Petar Antov, Abolfazl Soltani, Dorina Camelia Ilies, Elham Nadali, Seng Hua Lee, Vasile Grama, Tripa Simona

**Affiliations:** 1 Wood Science and Technology Department, Faculty of Materials Engineering &Interdisciplinary Sciences, Shahid Rajaee Teacher Training University (SRTTU), Tehran, 16788-15811 Iran; 2 Department of Mechanical Wood Technology, Faculty of Forest Industry, University of Forestry, 1797 Sofia, Bulgaria; 3 Department of Civil Engineering – Geotechnics, Faculty of Civil Engineering, Shahid Rajaee Teacher Training University (SRTTU), Tehran, 16788-15811 Iran; 4 Department of Geography, Tourism, and Territorial Planning, Faculty of Geography, Tourism, and Sport, University of Oradea, 410087 Oradea, Romania; 5 Department of Wood & Paper Science and Technology, Faculty of Natural Resources, University of Tehran, Karaj, 77871-31587 Iran; 6 Department of Wood Industry, Faculty of Applied Sciences, Universiti Teknologi MARA (UiTM) Cawangan Pahang, 26400 Bandar Tun Razak, Malaysia; 7 Laboratory of Biopolymer and Derivatives, Institute of Tropical Forestry and Forest Product, Universiti Putra Malaysia (UPM), 43400 Serdang, Malaysia; 8 Department of Textiles, Leather and Industrial Management, Faculty of Energy Engineering and Industrial Management, University of Oradea, 410058 Oradea, Romania

**Keywords:** Coating and finish, Paint pull-off strength, Porous structure, Sepiolite, Silver nano-suspension, Thermal modification, Biotechnology, Engineering, Materials science

## Abstract

Sepiolite is a silicate mineral that improves the fire properties in solid wood when mixed with a water-based coating. The present study was carried out to investigate and evaluate the effects of sepiolite addition to acrylic-latex paint on the pull-off adhesion strength, as an important characteristic of paints and finishes used in the modern furniture industry and historical furniture as well for preservation and restoration of heritage objects. Sepiolite was added at the rate of 10%, and brushed onto plain-sawn beech (*Fagus orientalis* L.) wood specimens, unimpregnated and impregnated with a 400 ppm silver nano-suspension, which were further thermally modified at 185 °C for 4 h. The results showed that thermal modification had a decreasing effect on the pull-off adhesion strength, primarily as a result of the thermal degradation of cell-wall polymers (mostly hemicelluloses). Still, a decreased wettability as a result of condensation and plasticization of lignin was also partially influential. Based on the obtained results,thermal modification was found to have a significant influence on pull-off adhesion strength. Sepiolite addition had a decreasing effectin all treatments, though the effect was not statistically significant in all treatments. The maximum and minimum decreases due to sepiolite addition were observed in the unimpregnated control (21%) and the thermally-modified NS-impregnated (4%) specimens. Other aspects of the sepiolite addition, and further studies that cover different types of paints and coatings, should be evaluated before coming to a final firm conclusion in this regard.

## Introduction

Utilization of different paints and finishes on wood surfaces is a common practice that has a long history, maybe as long as civilization itself. The reasons to apply a coating on wood surfaces can be categorized into two main groups. Firstly, wood coatings are applied as a protective measure against water absorption, physical damages like abrasion and scratch, fire, UV radiation, and sometimes chemicals; and secondly, they are used for aesthetic purposes to add to the beauty of the substrate. In many cases, combination of the above mentioned purposes from both categories is the case, satisfying protective and aesthetic purposes at the same time.

Apart from the purposes of wood coatings and finishes, the way a coating is stuck to the substrate and is retained on them over a particular time and under specific conditions is also of great importance for both the producers and the end-users. From an internal point of view and focusing on wood properties, great variations in biological factors, chemicals, porous system (including vessel, fiber, and tracheid properties), and the modification techniques that are commonly used to improve the inherent drawbacks in each wood species are influential in the outcome of applying different coats and finishes on each and every wood species. However, external factors like weathering and environmental conditions can also affect both wood and coating^1,2^. The combination of internal and external factors on the effectiveness of coatings and finishes is not only important in today's technological world of modern products, but it is also vital to choose the suitable preservation and conservation techniques for patrimony and heritage objects that are so delicate and should be maintained and preserved for future generations.

The existence of many wood species, and the variation in conditions under which a tree grows, can be considered the root cause for the occurrence of many differences not only among different wood species but among different stands of a single species as well. To improve homogeneity and uniformity among and within wood stands, engineers are constantly working to find new methods and techniques to modify wood species and to improve their properties as well. In this connection, thermal modification is considered an advanced method that has gained considerable success at a commercial scale^3,4^. Its environmentally-friendly aspects have also made it a safe modification technique widely used to enhance wood quality and prolong its utilization^5–13^. Increased dimensional stability, unification of surface colour, improved bio-deterioration resistance, and resistance to UV radiation are among the main advantages of this treatment^9,14–17^.

Although thermal modification may have negative effects on the strength properties of wood, some techniques have been developed to mitigate the negative effects^18^. In this connection, temperatures lower than 130 °C may only result in slight changes in wood properties, while temperatures higher than 230 °C result in undesired and mostly unacceptable degradation of the main wood cell wall polymers (cellulose, hemicelluloses, and lignin), and subsequent carbonization with the formation of CO_2_ and other pyrolysis products, making the heat-treated wood unsuited for the industry^9,19^. Thermal degradation starts with hemicelluloses, which are the most sensitive to heat wood cell wall components^9,20^. Degradation of hemicelluloses decreases the hygroscopicity of thermally modified wood, due to the reduced number of accessible hydroxyl groups^19,21,22^. The significant changes in the hygroscopicity of wood would significantly affect the pull-off adhesion strength of the woody substrate. Therefore, one of the main objectives of the present study was to find out the effects of thermal modification on the pull-off strength of beech wood.

As previously stated in this section, thermal modification at extremely low and high temperatures cannot result in suitable and desirable changes in wood properties. Moreover, the thermal conductivity of wood is relatively low, ranging from 0.1 to 0.2 W/mK^23^, probably making the outer shell of large wood pieces over-heated. Therefore, experiments were carried out to use metal nanoparticles with high thermal coefficients^24^ to prevent the accumulation of heat on the surface of wood pieces by accelerating its transfer to the inner parts. As a secondary objective, the impact of the improved thermal conductivity by impregnating beech specimens with nanosilver suspension was to be investigated.

Impregnation with metal nano-suspension, on the other hand, can cause changes in the porous structure of wood due to high pressure in vessels, as well as dissolving part of the extractives from cell cavities and vessels, which changes the fluid flow in wood^11,25^. These changes in the porous structure, as well as chemical composition, ultimately affect how paints and coatings penetrate into and adhere to wood substrates, resulting in a significant change in coating pull-off strength^26^.

In terms of the paint used, many additives can be easily dissolved and added to improve different properties in paints. Sepiolite is a fibrous hydrated Mg–silicate mineral (Mg_4_Si_6_O_15_(OH)_2_·6H_2_O) that has recently been successfully used to improve the fire properties in fir wood, both unheated and thermally-modified, and to improve thermal conductivity in oriented strand lumber (OSL)^27,28^. However, other aspects of sepiolite should be evaluated before it can be recommended on an industrial scale. One of these aspects is the pull-off strength of paints on solid wood species. Therefore, one of the main goals of the present study was to find out the effects of the addition of sepiolite to a commercial acrylic-latex paint. Moreover, as to the importance and popularity of thermal modification, a separate set of specimens was prepared to be first thermally modified, the results of which were compared with those of the unheated ones.

In terms of the paint and given the increasing popularity of acrylic-latex paints around the world, this type of paint was chosenfor the current study, despite the fact that thermal modification is well-known to negatively affect wettability in wood substrates^20^. However, additional research should be carried out to investigatesolvent-based paints, the use of a primer, or the use of an adhesion intermediate. As to the solid wood species, beech wood was chosen, as it is considered a well-known industrial wood species that is used in nearly all countries in many applications. The results of the present study could be useful for the furniture industry.

## Materials and methods

### Specimen preparation

**Figure 1:**
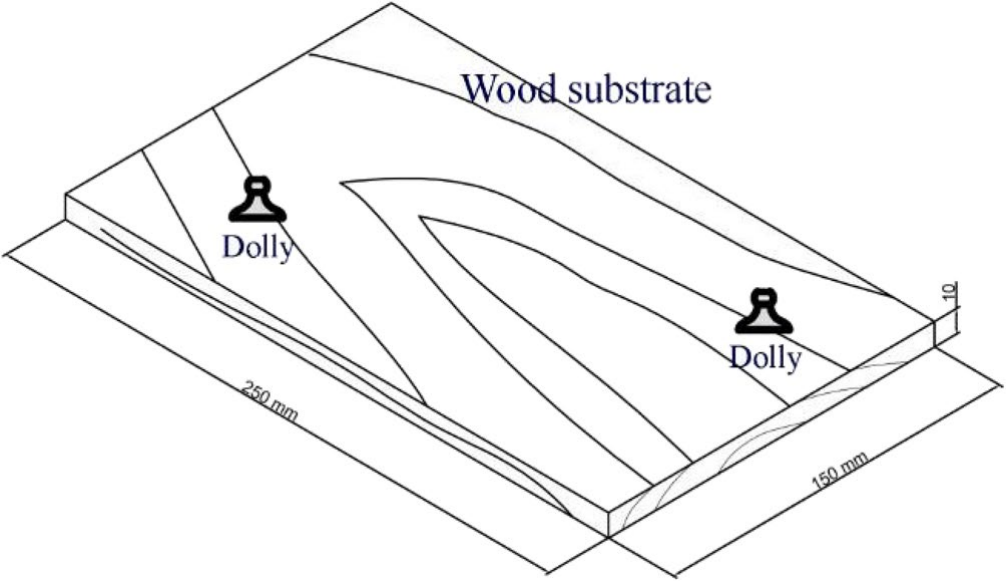
Schematic diagram of the plain-sawn specimens showing two dollies stuck on the upper surface.

Oriental beech (*Fagus orientalis* L.) is a wood species with medium density, having suitable properties to be used in the furniture industry. This species is naturally grown in the northern forests of Iran. Because of its widespread appeal among both woodworkers and final consumers, this material is in high demand. A total number of 48 board specimens were cut from plain-sawn beech timber. The dimensions of the board specimens were 250 mm (length) × 150 mm (width) × 10 mm (thickness). The mean density was measured to be 0.615 g cm^−3^ (moisture content of 9 ± 0.5%). The specimens were examined for knots, checks, fungal attacks, and other defects to ensure they were defect-free. The specimens were seasoned for two months at room temperature of 25 ± 2 °C and a relative humidity of 40 ± 3%. After conditioning, the wood surface was sanded with 100-grit sandpaper, followed by blowing air to remove the sanded surface's wood dust. Then, the samples were brushed with a water-based acrylic-latex paint. Once coated, they were again kept in the conditioning chamber for two more weeks. Using epoxy resin, two dollies were stuck to the painted surface of each board specimens prior to performing the pull-off test (Fig. 1). This way, eight replicate dolly specimens were prepared for each of the twelve treatments. The moisture content of wood specimens was 9 ± 0.5% at the time of pull-off tests.

Acrylic paints have become popular in wood products^29^ over the last two decades. Therefore, a water-based acrylic-latex paint (code number ALCO-6510) was used in the present study, purchased from Alvan Paint and Resin Production Co. (Tehran, Iran). The solid content of the paint was reported to be 37% ± 1%. The paint was brushed on the specimens twice, with a two-day interval between the two coats. No sanding was done between the two coats, or afterwards. The final thickness of the coating was 200 ± 30 μm when dried. Once coated, the samples were kept in a conditioning chamber for two weeks at a temperature of 25 ± 2 °C and 40 ± 3% relative humidity.

**Figure 2:**
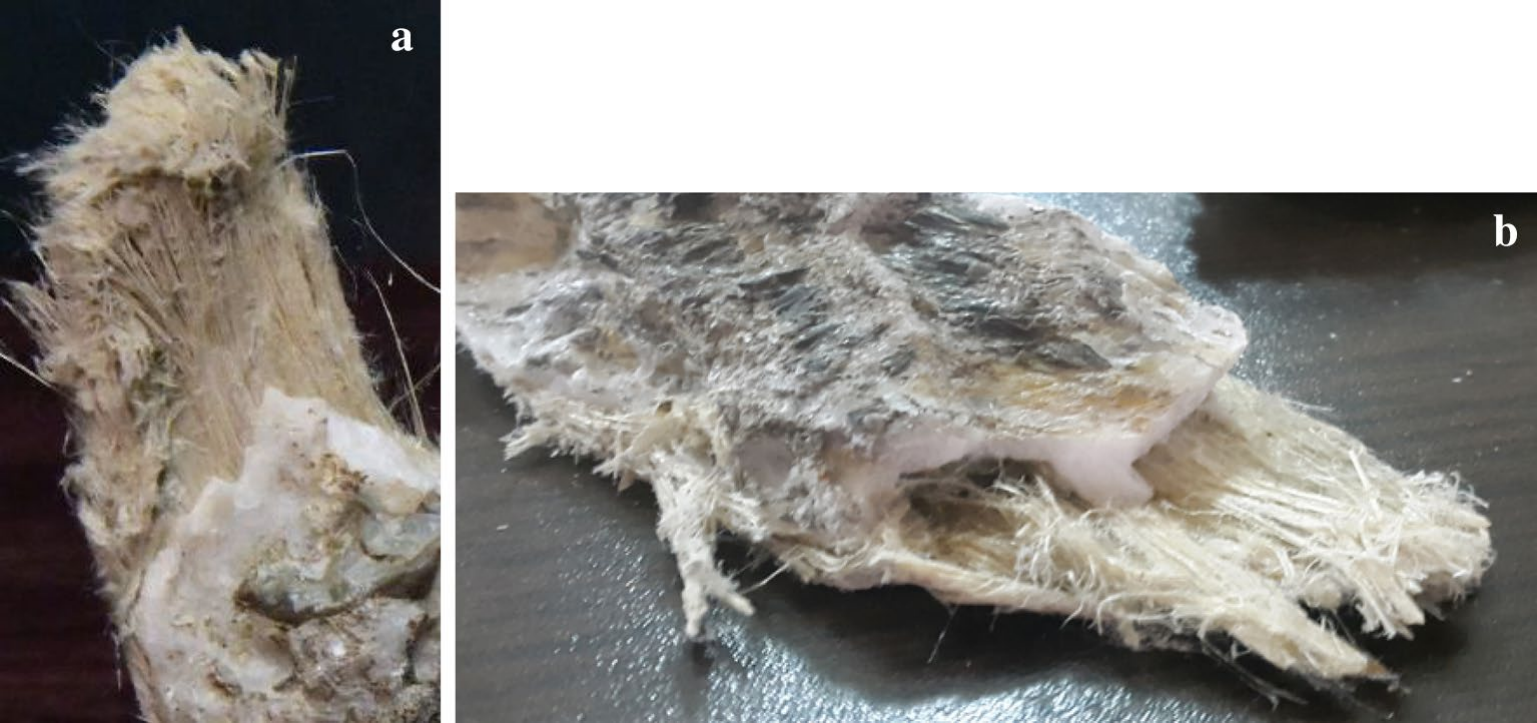
Photos showing microfibers in sepiolite (a and b).

**Figure 3:**
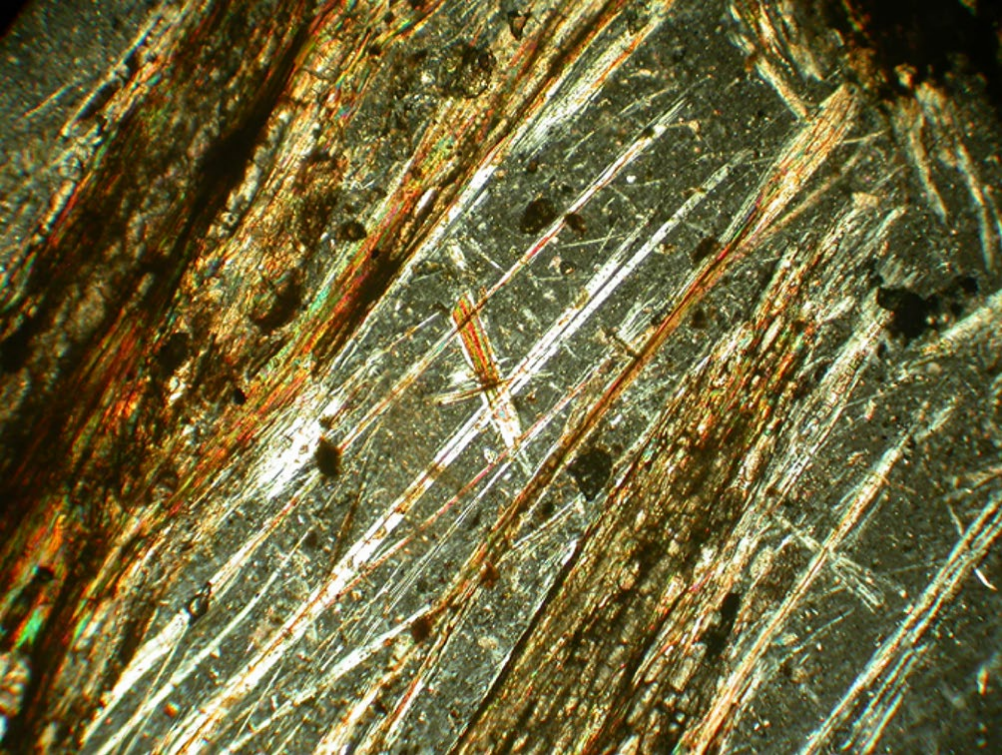
Photomicrograph of sepiolite microfibers taken by polarized microscope (cross-polarized light XPL, × 72).

**Figure 4:**
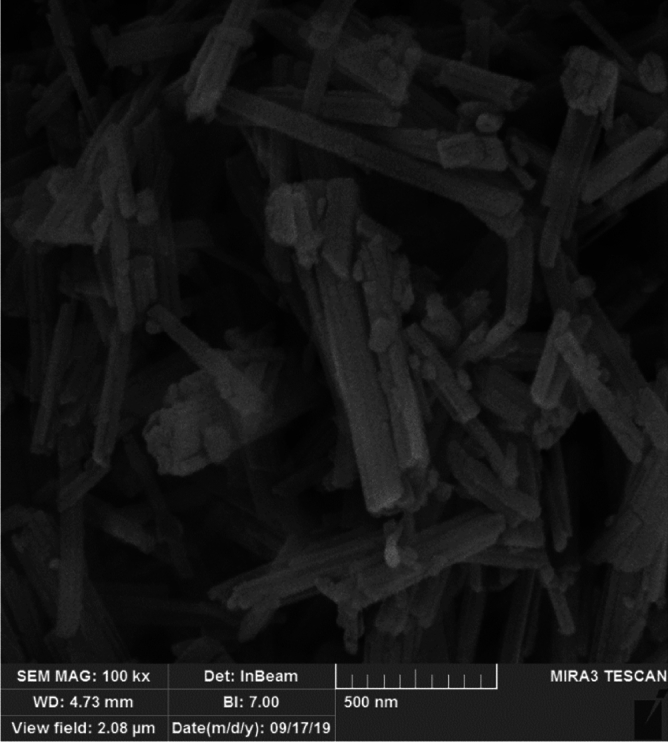
SEM image of sepiolite microfibers.

For the sepiolite-treated coating, 10% dried sepiolite dry powder (based on the wet weight of the paint) was added to the acrylic paint before being brushed onto the specimens. The 10% was chosen based on a previous study on sepiolite used as a fire-retardant^28^. The sepiolite mineral was extracted from the Tanbu region, South Minab, Iran. It is texturally recognized by long fiber crystals (up to 60 cm long, with a ratio of length to width > 1200). They are associated with calcite, dolomite, serpentinite, and talc minerals^30–32^. The fibers are light yellow-to-greenish color in hand specimens and vary in texture such as blades, strands, and laminated clusters (Fig. 2a,b). To observe the structure of sepiolite fibers, five thin sections were prepared and studied using a binocular polarized microscope (model: LeitzLaborlux 12 POL, Leitz, Germany) available at the Faculty of Civil Engineering Lab (SRTTU, Tehran, Iran). Figure 3 represents a combination of clustered and needle-like (acicular) crystals of Tanbu sepiolite. Using a Los Angeles abrasion machine (H-3860D, Humboldt Mfg., USA) the purified sepiolite fibers were milled until a micro-scale homogenized powder was obtained. The machine was equipped with 12 steel balls and ground fibers for three hours confirming the standard test method (ASTM C 131)^32,33^. To increase the specific surface area of sepiolite, the process of grinding of micronized powder was continued using an agate pestle (mortar) until the nanoparticles of sepiolite fibers were obtained, as can be observed in SEM images (Fig. 4).

### Impregnation with silver nano-suspension

The silver nanoparticles were dispersed in water at 400 ppm. The aqueous nano-suspension was produced using an electrochemical technique for impregnating specimens^34^. At least, 90% of the particles ranged from 30 to 80 nm. Based on the producer’s report, the formation and size of the silver particles in the nano-suspension were monitored by the well-known transmission electron microscopy, and checked by drop-coating onto carbon-coated copper grids. Beech specimens were impregnated in a pressure vessel, using the empty-cell process (Rueping method). The impregnation was carried out at Mehrabadi Machinery Mfg. Co. Ltd. (Tehran, Iran). The pressure was set at 300 kPa for 30 min. Before and after the process of impregnating, all specimens were weighted to the accuracy of 0.01 g, with a digital scale, so that the nano-suspension uptake could be measured in the specimens. Once impregnated, they were kept in a conditioning chamber (temperature of 25 ± 2 °C, and relative humidity of 40 ± 3%) along with all the other specimens for two months.

### Thermal modification process

Specimens for thermal modification (at 185 °C) were randomly arranged in the middle tray of a laboratory oven, with the upper and lower trays kept empty, to ensure uniformity of thermal modification among the specimens. Thermally-modified specimens were marked with TM, and specimens impregnated with nanosilver were coded as NSI. The coding system used in the present project is summarized in Table 1. Specimens were placed on 3 mm thick wood strips to prevent direct contact with the metal tray in the oven which could lead to overheating at the points of direct contact. In order to comply with previous studies for further comparison, specimens were first thermally modified at 145 °C for twelve hours, followed by four hours at 185 °C. TM and NSI-TM specimens were simultaneously thermally modified. All samples were then moved to a conditioning chamber (25 ± 2 °C, and 40 ± 3% relative humidity).

**Table 1:** Description of the coding for the thermal modification and impregnation treatments performed in this research work.

Coding	Description of the treatment
Control	Specimens with no impregnation and modification
Control-NSI	Specimens impregnated with silver nano-suspension
TM185	Thermally-modified specimens at 185 °C
NSI-TM185	Nanosilver-impregnated specimens, thermally modified at 185 °C

### Pull-off adhesion strength measurement

**Figure 5:**
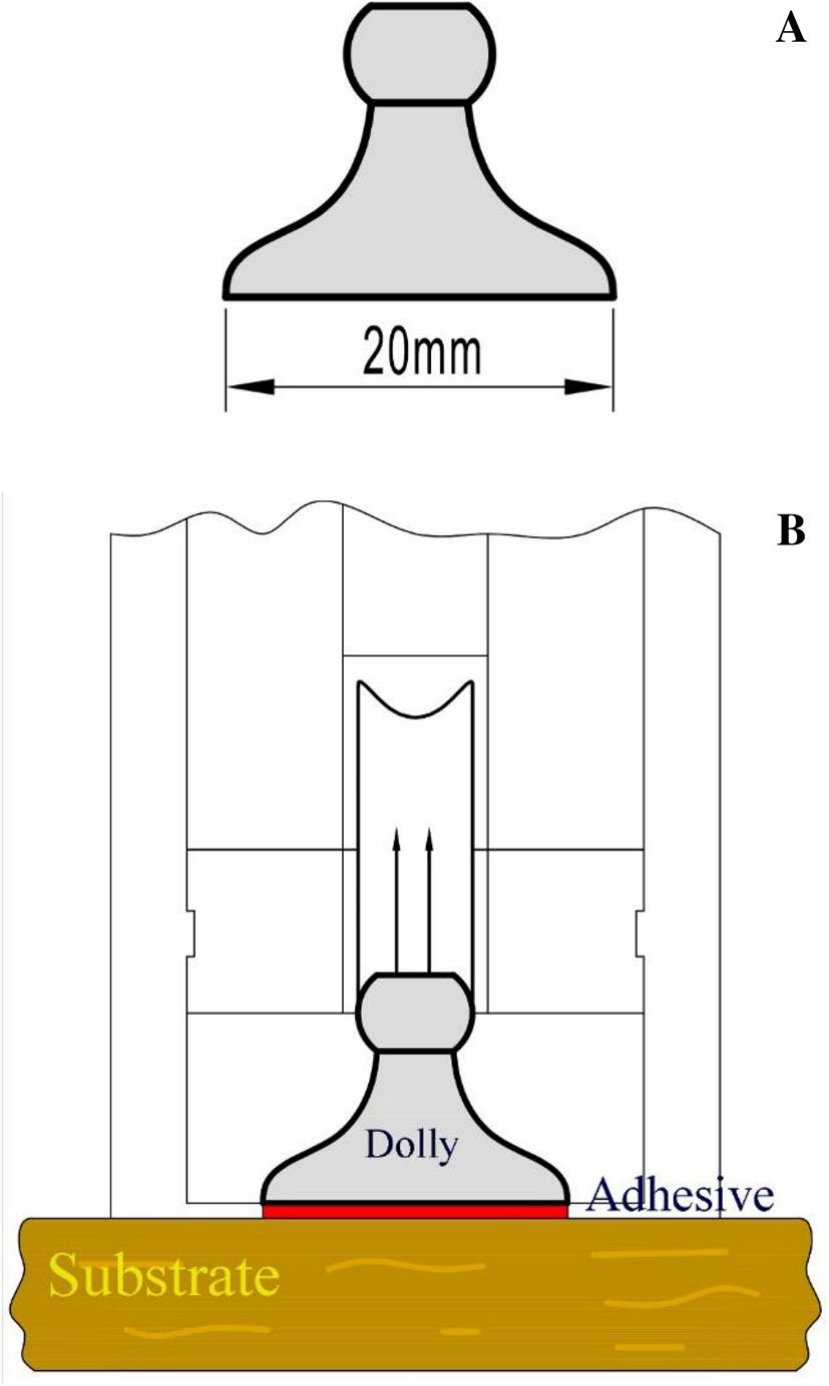
Schematic design of the dolly to be stuck to the plain-sawn beech wood substrate; (**A**) dolly with an effective surface diameter of 20 mm, (**B**) dolly stuck to the plain-sawn wood specimen.

Pull-off adhesion strength measurement is characterized by the maximum strength required to break the circular surface of a dolly that is stuck to a specimen (the substrate). In the present study, the effective surface of the dolly was 1256 mm^−2^ (Fig. 5). Five plain-sawn specimens were prepared for each treatment. The dimension of the samples was 250 mm × 150 mm × 15 mm. Two dollies were stuck to the substrates (the samples) with epoxy resin and allowed to polymerize for 24 h. Pull-off adhesion strength was then measured, using an automatic PosiTest® apparatus (Defelsko, Og-densburg, NY, USA), and in accordance with the specifications in ASTM D 4541-02 standard(2006)^35^. Once the maximum force was measured in each specimen, the pull-off strength (*X*) was calculated (in MPa), using Equation No. 1. The breaking surface was checked in each specimen to make sure that failures occurred in the body of the substrate. Based on the standard specifications, the results were eliminated if the failure occurred in the adhesive layer.

1 $$X = \frac{4\;F}{{\pi \cdot d^{2} }}$$

where *F* represents the maximum force at the failure point (kg m s^−2^), and *d* stands for the diameter of the dolly (mm), based on ASTM D4541-02 testing specifications (2006)^35^. The moisture content of the specimens was monitored to be 9 ± 0.5%, when the pull-off adhesion tests were carried out, and the temperature was measured to be 25 ± 3 °C.

### Tangential gas permeability

Different procedures were initiated to measure permeability and diffusion in wood, lignocellulose-based composite panels, and other porous media^36,37^. In the present study, tangential gas permeability was measured by an electronic apparatus with millisecond precision, using the falling water method (USPTO 8079249 B2, 2011)^38^. Ten replicate specimens of 17.5 mm in diameter and 5 mm in thickness were cut in tangential direction of the four main treatments without the paint (control, NS-impregnated, thermally-modified, and NS-impregnated thermally-modified treatments), usingahollow circular saw. All around the specimens were covered with silicon adhesive to prevent airflow from undesired directions. The specimens were then fixed in the holder of the apparatus, using silicon hoses to ensure that the whole structure was air-tight. The superficial gas permeability coefficient was calculated by the equations presented by Siau^39,40^, to be further modified by a correction factor to obtain the specific tangential gas permeability.

### Scanning electron microscopy (SEM imaging)

A TESCAN-Mira 3 apparatus (TESCAN, Brno, Czech Republic) was used to carry out the SEM imaging. The apparatus was at the thin-film laboratory, FE-SEM lab (Field Emission), School of Electrical & Computer Engineering (The University of Tehran, Tehran, Iran). A field-emission cathode in the electron gun of the SEM provided narrower probing beams at both low and high electron energy levels. This provided an improved resolution, and lower damage to the sample because of minimized charging.

### Statistical analyses

Three-way analysis of variance (ANOVA) was conducted to discern for statistical analysis at a 95% level of confidence. The analysis was carried out using SAS software program (version 9.2) (2010). To distinguish the statistical difference among the categories, the Duncan multiple range test was conducted.

## Results

The control beech specimens had a mean density of 0.615 ± 0.056 g cm^−3^. After being impregnated with NS-suspension, the specimens were removed from the pressure vessel, and their weight was immediately measured using a digital laboratory scale. The average NS-suspension uptake was determined to be 0.39 ± 0.042 g cm^−3^.

**Figure 6:**
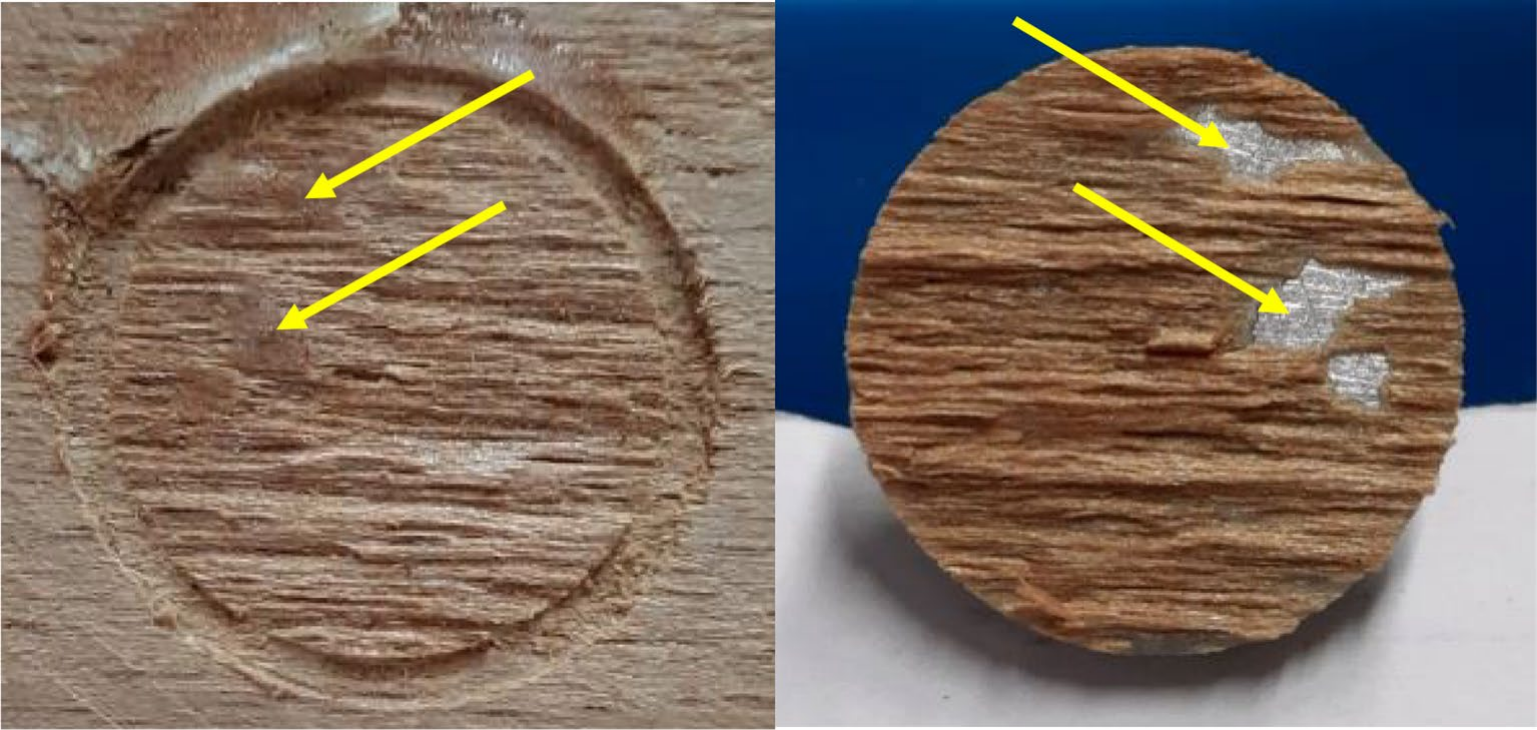
Pull-off specimen of the control beech substrate showing remnants of the adhesive portions where the adhesive layer is visible in the probably irregular areas of the specimen (left image ↓), and the aluminum surface of tested dolly demonstrating that the failure partially occurred in the adhesive layer (right image ↓) (magnification × 2.5).

**Figure 7:**
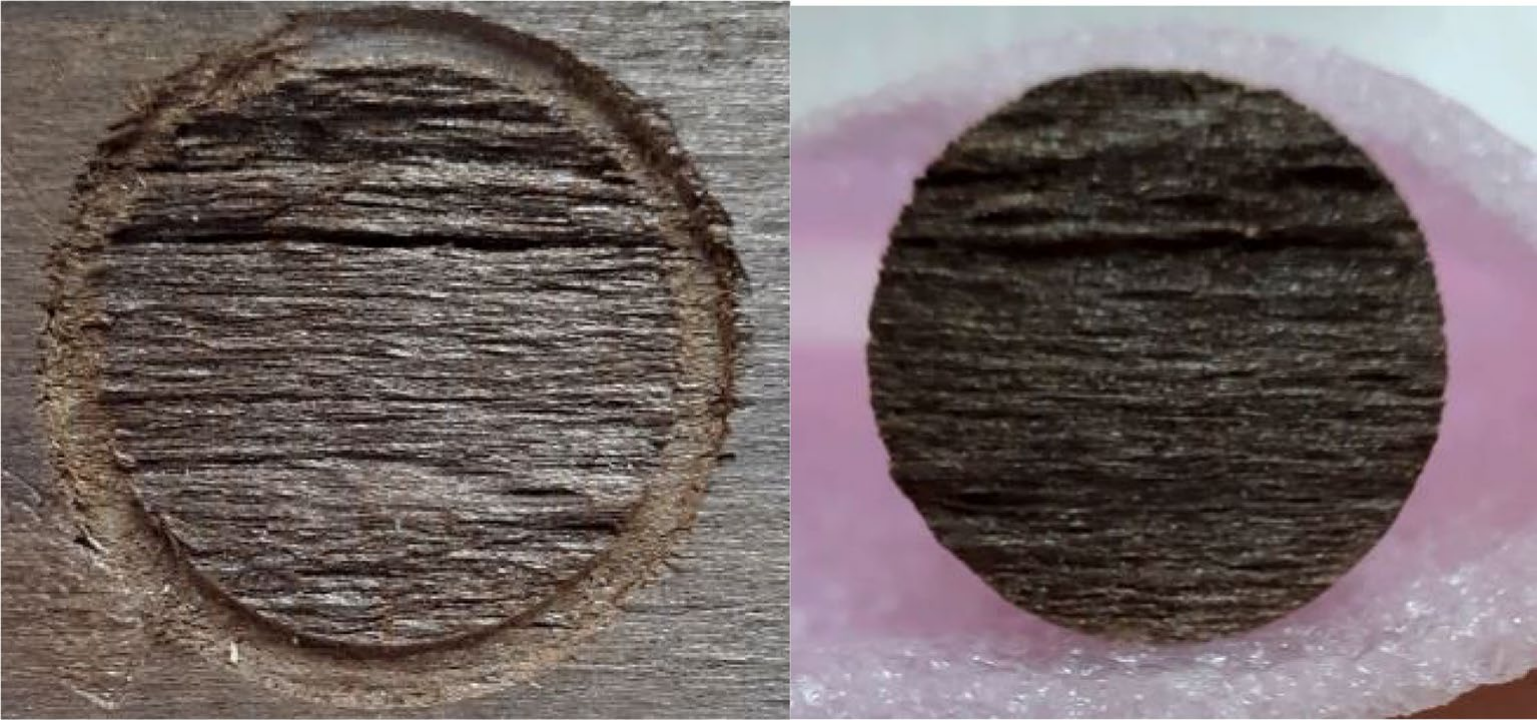
Pull-off specimen of the thermally-modified beech substrate illustrating full and accepted failure in the body of the wood substrate (no failure occurred in the adhesive layer) (magnification × 2.5).

The breaking surface was checked after each dolly was tested. Figure 6 depicts a specimen in which the adhesive layer failed, and thus the test result was not included in the mean calculation. The complete failure of the circular part of the body of the wood substrate indicated that the test result was acceptable (Fig. 7).

**Figure 8:**
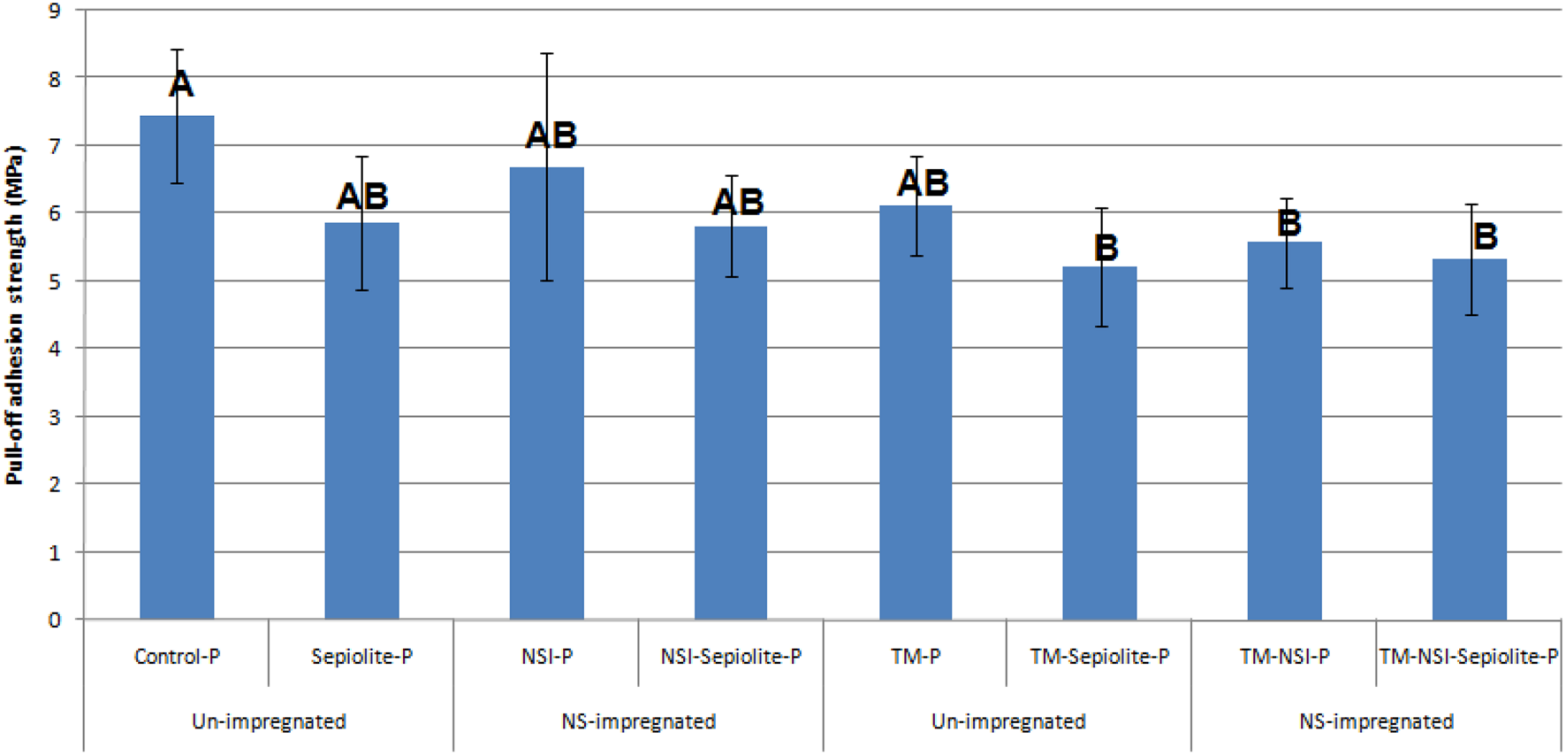
Pull-off adhesion values (MPa) of plain-sawn beech specimens, painted with an acrylic-latex paint (*P* painted, *NSI* nanosilver-impregnated, *TM* thermally-modified specimens). Error bars represent the standard deviation; letters on each column represent Duncan's multiple range groupings based on a 95% level of confidence.

The maximum and minimum pull-off strength values in the painted specimens were found in the Control-P (7.45 ± 0.99 MPa) and TM-Sepiolite-P (5.21 ± 0.87 MPa), respectively (Fig. 8). Impregnation with silver nano-suspension reduced pull-off strength values in nearly all treatments, though the decrease was not statistically significant in some cases. Furthermore, the addition of sepiolite to the paint reduced the pull-off strength in all treatments, though the decreases were not statistically significant in most of the cases.

**Figure 9:**
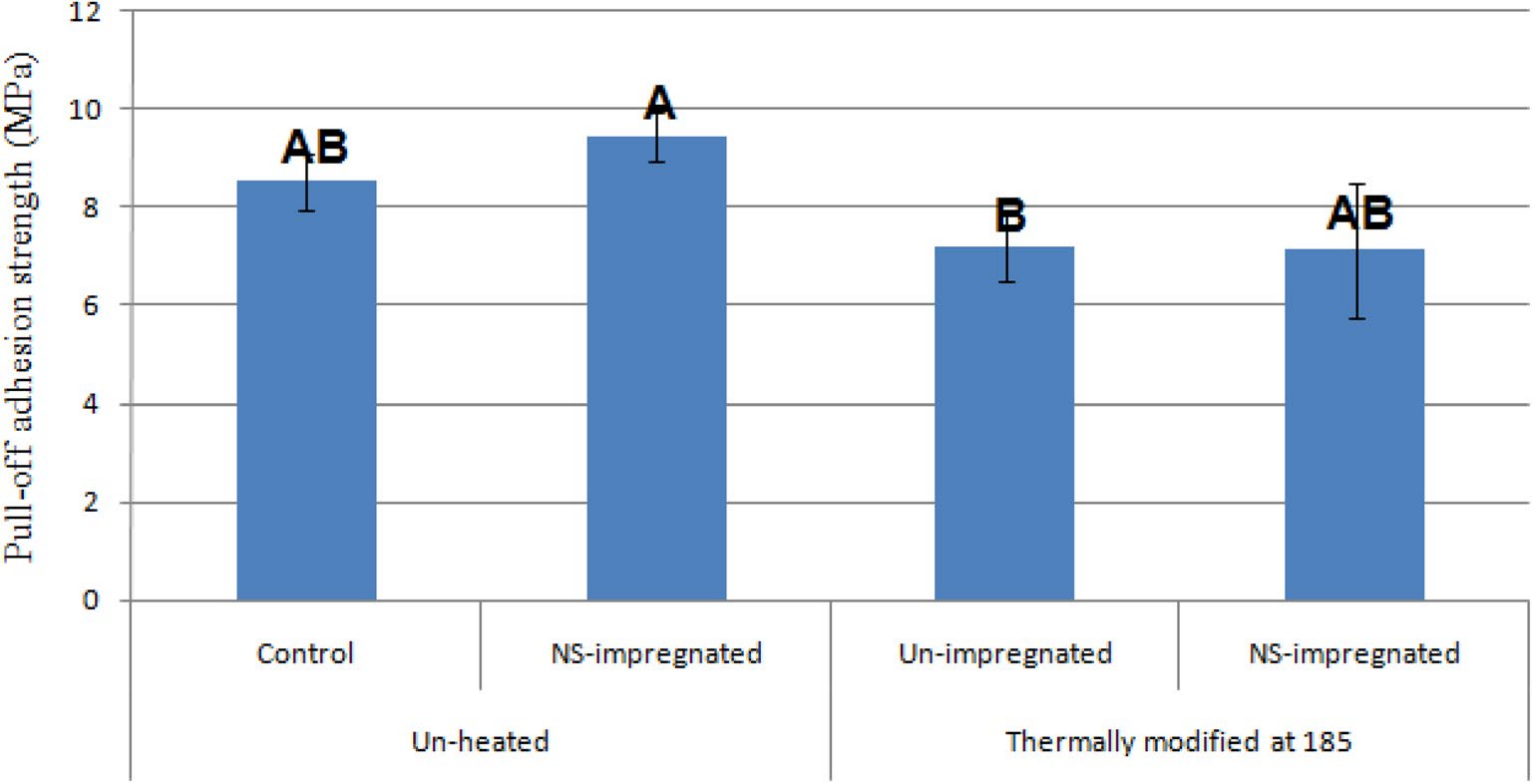
Pull-off adhesion values (MPa) of plain-sawn beech specimens, without any finish or paint (*NS* nanosilver). Error bars represent the standard deviation; letters on each column represent Duncan's multiple range groupings based on a 95% level of confidence.

All pull-off strength values, determined in the unpainted treatments, were higher in comparison to their painted counterparts (Fig. 9). Impregnation with NS-suspension increased pull-off strength in the unpainted unheated specimens by 10%, while in the heat-treated ones, the value statistically remained the same. The highest value in the unpainted treatments was observed in the unheated NS-impregnated specimens (9.45 MPa).

Thermal modification decreased pull-off strength in all treatments, although the decreased values were not statistically significant compared to the unheated counterparts (Figs. 8 and 9). NS-impregnation showed no significant impact on the pull-off strength in the thermal modification of specimens.

The results of the permeability measurement revealed that the mean tangential gas permeability was 0.0008 (× 10^–13^ m^3^ m^−1^). The use of NS-suspension increased permeability by 25%. Thermal modification increased the permeability of the unimpregnated and NS-impregnated specimens by 152% and 185%, respectively.

## Discussion

**Figure 10:**
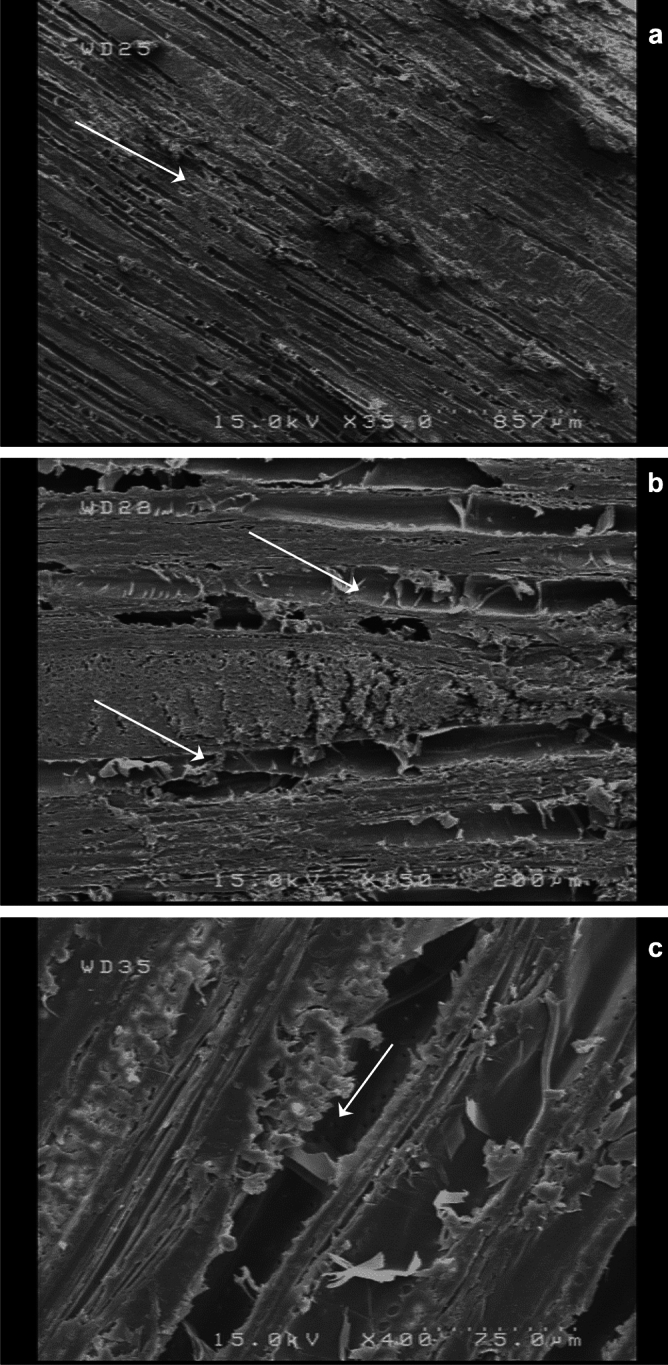
SEM images (**a**–**c**) showing the vessel elements and fiber cavities (↓) with different magnitudes in the plain-sawn beech wood.

The highest pull-off adhesion strength values were found in the unpainted treatments, both in the unheated specimens, and the heat-treated ones (Figs. 8 and 9). This was attributed to the fact that in the unpainted treatments, vessel elements and cell cavities were open and not covered with paint (Fig. 10a–c); therefore, the resin that stuck the dollies to wood substrates could easily penetrate the wood texture (vessels’ and cells’ cavities), providing a much better anchoring between dollies and wood. Moreover, and considering the fact that all failures occurred in the wood substrate of the specimens, the higher pull-off adhesion strength values in the unpainted implied that the anchoring of the epoxy resin was more effective in comparison to the water-based paint that was used. The depth of paint penetration into the texture of the wood substrate was not part of the evaluation in the present study, nor was the measurement of the thickness of wood failure that was stuck to the dollies, once tests were completed. Therefore, to come to a firm conclusion on the effects of different paints and coatings on anchoring, further studies should be carried out.

The addition of sepiolite to the paint resulted in a decrease in the pull-off adhesion strength of all treatments, though in some cases the amounts of decrease were not statistically significant (Fig. 8). In previous studies, the addition of another silicate mineral, namely wollastonite, to some resins, like urea formaldehyde (UF) and poly-vinyl acetate (PVAc), was reported to have contradictory effects; that is, the addition resulted in both improvement and weakening of the resin strength^37,38^, depending on the resin type and content, and the evaluated mechanical property. Wollastonite acted as a reinforcement agent or extender in the PVAc matrix, improving the shear strength of the resin to stick unheated and heat-treated solid wood species^41^. In oriented-strand lumbers (OSL) containing 8% UF resin, the addition of wollastonite resulted in a decrease in some mechanical properties, including the modulus of rupture (MOR), modulus of elasticity (MOE), internal bond (IB), tension parallel to the grain, and shear strength^42^. However, increasing the resin content to 10% resulted in an improvement in all the above-mentioned properties. According to the cited authors, wollastonite serves two purposes. It primarily served as a reinforcing agent in the resin, thereby increasing its strength. Furthermore, it formed new bonds with cell-wall polymers (primarily cellulose and hemicelluloses), resulting in increased resin strength. Based on the above-mentioned previous studies, and considering the decreasing effect of sepiolite in the present study, it can be concluded that the addition of 10% sepiolite gel to water-based paints results in a slight decrease in pull-off adhesion strength. However, this decreasing effect cannot be generalized to all silicate minerals (like sepiolite and wollastonite), all paints and finishes, and different silicate content levels as well. In order to come to a final conclusion that can be representative of how the addition of sepiolite works, a wider range of different paints and finishes (with different types of solvents like water and organic), and silicate content levels, should be evaluated.

As mentioned in the Results section, impregnation with nanosilver suspension primarily increased pull-off adhesion strength in the unpainted unheated specimens by 10% (Fig. 9). This increase, although statistically not very significant, was attributed to two main reasons based on previous studies on solid wood species^26,43^. The first reason was attributed to the low concentration of NS-suspension (only 400 ppm). The aqueous nano-suspension dissolved water-soluble extractives like simple sugars, starch, lignans and other phenolic compounds from the specimens^26^. The second reason is the distortion of vessel perforation plates and pit torus, caused by the pressure vessel while the specimens were being impregnated. Both of these increased permeability which in turn resulted in a more effective anchoring of paint on the substrate. In another study that measured pull-off strength in longitudinal direction (pull-off values in the cross-section of specimens) in beech wood specimens, a decrease was reported^44^. The authors reported that a similar increase in permeability resulted in a decrease in pull-off strength. In this connection, it should be noted that permeability is substantially higher in the longitudinal direction in hardwood species^39,40,43^. It was therefore concluded that the positive or negative effects of an increase in permeability on pull-off strength are significantly dependent on the direction of wood. That is, while an increase in the permeability of plain-sawn wood species can positively affect the pull-off strength, an increase in the permeability in the cross-section direction can negatively affect the pull-off value, because the resin can easily penetrate the vessel element, making it out of reach to be practically involved in the process of sticking the dolly to the wood substrate.

On the other hand, in the painted specimens the impact of impregnation with nanosilver suspension was decreasing, though no statistical significance was observed between the unimpregnated and nanosilver-impregnated specimens. In this connection, more studies on different paints and finishes should be carried out before a firm conclusion is made in this regard.

All heat-treated treatments demonstrated lower pull-off strength adhesion values in comparison to their unheated counterparts, though the difference between the pairs was not statistically significant in all cases. The general decrease in all heat-treated treatments is attributed to multiple factors. The first and probably the most important factor is the thermal degradation of cell-wall polymers (mostly cellulose and hemicelluloses) as a result of thermal modification^4,5,7,9–12,20,45^. The decreasing trend in the pull-off strength of the thermally-modified specimens is consistent with previous studies, though the type of finishes and paints would be influential in the final results of pull-off strength^44^. Other coating properties were also reported to decrease as a result of thermal modification, including coating hardness, and scratch resistance, in different wood species like iroko, chestnut, limba, and ash^46^. Condensation of lignin is another factor that causes a decrease in the mechanical properties of solid wood, partially influential in the decrease in the pull-off strength of the heat-treated specimens^5,47^. Thinning of the cell wall, and the occurrence of microscopic cracks in it, is also a well-known side-effect of condensation of lignin and thermal modification^4,5,7,9,11,12,20,45^ having a deteriorating effect on pull-off adhesion strength values.

## Conclusions

Thermal modification generally demonstrated a decreasing effect on the overall pull-off adhesion values. This was primarily attributed to the thermal degradation of cell-wall polymers (mainly hemicelluloses), having a decreasing impact on the overall mechanical strength of the modified wood specimens. However, the decreased wettability as a result of plasticization and condensation of lignin intensified the decreasing impact. The addition of sepiolite had an insignificant decreasing effect on the pull-off adhesion strength. Therefore, it was concluded that the addition of sepiolite to acrylic-latex paint to improve pull-off adhesion strength cannot be recommended. However, in order to come to a final decision on other aspects of the addition of sepiolite in wood paints and coatings, other test methods for paints and coatings should also be carried out (like cross-cut test method together with the TABER method, ASTM D 4060 Standard Test Method for Abrasion Resistance of Organic Coatings). Moreover, other aspects of the addition should also be considered, like probable improvement of fire properties, and resistance to deteriorating fungi, insects, and termites. Further studies should be carried out in which different levels of silicate-based minerals (like sepiolite and wollastonite) are added to a variety of paints and coatings with water and organic solvents, in order to investigate important coating properties, such as pull-off adhesion strength, scratch, and UV resistance, etc. The results obtained can have multiple functions and could provide valuable information about the feasibility of using silicate-based materials in wood-based industry or for the preservation and restoration of historical and cultural heritage objects, so that these priceless objects can be preserved for future generations.

### Supplementary Information

Supplementary Information.

## Data Availability

The datasets used and/or analyzed during the current study are available from the corresponding author upon reasonable request.
